# Recurrence Networks in Natural Languages

**DOI:** 10.3390/e21050517

**Published:** 2019-05-23

**Authors:** Edgar Baeza-Blancas, Bibiana Obregón-Quintana, Candelario Hernández-Gómez, Domingo Gómez-Meléndez, Daniel Aguilar-Velázquez, Larry S. Liebovitch, Lev Guzmán-Vargas

**Affiliations:** 1 Departamento de Física, Escuela Superior de Física y Matemáticas, Ciudad de México 07738, Mexico; 2 Unidad Profesional Interdisciplinaria en Ingeniería y Tecnologías Avanzadas, Instituto Politécnico Nacional, Ciudad de México 07340, Mexico; 3 Facultad de Ciencias, Univesidad Nacional Autónoma de México, Ciudad de México 04510, Mexico; 4 Unidad Académica de Ingeniería Eléctrica, Universidad Autónoma de Zacatecas, Zacatecas 98000, Mexico; 5 Department of Physics, Queens College, City University of New York, New York, NY 11367, USA; 6 Advanced Consortium on Cooperation, Conflict, and Complexity (AC4), Earth Institute, Columbia University, New York, NY 10027, USA; 7 Graduate Center, City University of New York, New York, NY 10016, USA

**Keywords:** recurrence networks, natural languages, patterns repetition

## Abstract

We present a study of natural language using the recurrence network method. In our approach, the repetition of patterns of characters is evaluated without considering the word structure in written texts from different natural languages. Our dataset comprises 85 ebookseBooks written in 17 different European languages. The similarity between patterns of length *m* is determined by the Hamming distance and a value *r* is considered to define a matching between two patterns, i.e., a repetition is defined if the Hamming distance is equal or less than the given threshold value *r*. In this way, we calculate the adjacency matrix, where a connection between two nodes exists when a matching occurs. Next, the recurrence network is constructed for the texts and some representative network metrics are calculated. Our results show that average values of network density, clustering, and assortativity are larger than their corresponding shuffled versions, while for metrics like such as closeness, both original and random sequences exhibit similar values. Moreover, our calculations show similar average values for density among languages which that belong to the same linguistic family. In addition, the application of a linear discriminant analysis leads to well-separated clusters of family languages based on based on the network-density properties. Finally, we discuss our results in the context of the general characteristics of written texts.

## 1. Introduction

During pastIn recent decades many studies have pointing pointed out the complexity and organizational properties of natural languages, specially especially for written texts. These studies have attracted the attention of researchers from different areas of science who use different approaches to tackle the complexity of natural languages [1,2,3,4,5,6]. From a morphological point of view, languages are classified in analytics and synthetics. The key difference between one and othersthem is the use of the forms of a lexeme to build words and sentences. In this context, many methods developed in the study of time-series analysis and network science are potentially suitable to evaluate this kind of structural differences [7,8,9,10,11,12,13,14,15].

Of particular importance are methods based on based on the quantification of recurrence features from systems whose phase phase-space representation may provide information about invariant properties of particular configurations [11]. For instance, the graphical representation of the recurrence plots permits to visually identifyvisual identification of specific signatures of periodicities or cycles, and these signatures can be quantified in terms of fundamental invariant properties of a dynamical system [16]. In this context, Marwan et al. [17] introduced the concept of recurrence network, as an extension of the recurrence plot analysis, where the recurrence matrix is used to obtain the adjacency matrix, which contains the information of the connectivities. The characterization of the recurrence networks provides additional information of to that obtained from the standard recurrence quantification analysis and permits a direct evaluation of the relatedness of vectors defined in an *m*-dimensional space [18].

The study of language from a dynamical perspective have has been addressed by researchers from different areas of science, ranging from natural natural-language processing to network and information theory [19,20,21,22]. For instance, distributions of distances between successive occurrences of a specific word are well described by a stretched exponential, indicating the presence of memory [23], while other studies have considered the recurrence of words within sentences or paragraphs to estimate the correlation dimension of sets of paragraphs (discourse) [24].

We are interested in evaluating the recurrence of patterns (sequences of characters) of a given length which may provide an alternative approach to look into the differences and similarities, between different natural languages. In particular, we resort to methods like such as correlation dimension and recurrence networks for a direct quantitative way of measuring the level of recurrence with respect to the random configuration of the texts [11,25]. Our goal is to analyze the recurrence of patterns along the text, by examining the spatio-temporal organization of these patterns from a network science perspective. Recently, we reported the application of methods like such as the approximate entropy to the study of irregularities displayed by some natural languages [26,27,28]. In our approach, we consider the similarity between two patterns of length *m* based on based on the Hamming distance among them. We define a distance (Hamming distance *h*) and define a “matching” between two patterns if the distance is equal or less than a given value *r*. In this way, we are able tocan construct the corresponding adjacency matrix, where a connection between two nodes (patterns) exists when a matching occurs. The recurrence network is constructed for texts from different European languages and some representative network metrics are calculated. First, we study the scaling behavior between the density of the network, a measure closely related to the correlation dimension, and the Hamming distance. Our calculations of several network metrics show similarities between languages which belong to the same linguistic family and differences among the linguistic families as well. These similarities and differences explored here have not yet been reported by any other methodology based on based on nonlinear dynamics. We also compare the values of these network metrics of actual data with their corresponding randomized versions. The paper is organized as follows. In Section 2, the method to construct the recurrence networks and the dataset areis described. The results and discussion are presented in Section 3. Finally, some concluding remarks are given in Section 4.

## 2. Methods

### 2.1. Recurrence Networks

One of the characteristics frequently observed in dynamical systems is the recurrence of states, identified as states or configurations which become close to previous ones after some time [11,17]. The Hamming distance *h* is the number of distinct characters when two patterns of the same length *m* are compared. In our approach, we consider a text with length *N* and construct n=N-m+1 subseries (patterns) $x_{i}^{m}$ of length *m*. Next, we compare each of these patterns one by one and establish a matching if the Hamming distance is less than or equal to a tolerance parameter *r*. More formally, these recurrences are used to construct a recurrence matrix, defined as:

(1) $$R_{ij}=\Theta \left(r-h\left(x_{i}^{m},x_{j}^{m}\right)\right),$$

where Θ(-) represents the Heaviside function and $h(x_{i}^{m},x_{j}^{m})$ the Hamming distance. The adjacency matrix associated with the recurrence network is given by $A_{ij}=R_{ij}-\delta_{ij}$, with $\delta_{ij}$ the Kronecker delta. Thus, each pattern represents a node, and a connection (link) is defined if there is a match. For instance, in Table 1 we show a simple example of the matrix $R_{ij}$ for the Hamlet’s soliloquy: *To_be_or_not_to_be*. In this example, the threshold value r=2 is used to illustrate the construction of the recurrence matrix. In general, it is expected that for very regular texts, the number of repetitions subjected to tolerance *r* would be bigger compared to the case of a very irregular sequence of symbols where a matching is quite difficult unlikely to occur.

### 2.2. Network Metrics

During past yearsIn recent years, many studies focused on complex systems have used the network approach to characterize spatial and temporal organization of systems composed of interacting units [29]. Network measures may be useful to understand diverse properties of large sequences of symbols, which are transformed to matrices like like in the case of recurrence analysis [17,18]. Here, we listed some basic network metrics:

- Density (ρ): The density of a network is defined as:
  (2) $$\rho =\frac{2g}{n(n-1)},$$
  with *g* the number of actual connections and *n* is the number of nodes (patterns). A value of ρ close to 1 denotes an almost complete graph and ρ close to 0 indicates a poorly connected network.
- Closeness centrality ($K_{c}$): Measures the centrality of a given node in the network, defined as the reciprocal of the sum of the length of the shortest paths between the node and all other nodes in the graph [29],
  (3) $$K_{c}=\frac{n}{\Sigma_{j}d_{ij}},$$
  where $d_{ij}$ denotes the distance from node *i* to node *j*.
- Clustering coefficient ($C_{i}$): Measures the degree of transitivity in connectivity amongstamong the nearest neighbors of a node *i* [29]. In recurrence terms, $C_{i}$ represents the extent to which neighbors of a node (pattern) *i* are also recurrent amongstamong themselves. Specifically, $C_{i}$ is given by,
  (4) $$C_{i}=\frac{2E_{i}}{k_{i}\left(k_{i}-1\right)},$$
  where $E_{i}$ is the number of links between the $k_{i}$ neighbors of the node *i*.
- Average nearest-neighbor degree (${\bar{k}}_{nn,i}$): This measure allows us to see the mean preference in connectivity of a given node [30,31,32]. The behavior of this quantity as a function of the node’s degree, reveals whether high-degree nodes connect with other equally high-degree ones (assortativity), or high-degree nodes preferentially connect to low-degree ones (dissortativity) [29]. For unweighted networks, ${\bar{k}}_{nn,i}$ is calculated as:
  (5) $${\bar{k}}_{nn,i}=\frac{1}{k_{i}}\sum_{j=1}^{N}A_{ij}k_{j},$$
  where $k_{i}$ is the node’s degree, $A_{ij}$ represents the adjacency matrix and *N* is the number of nodes.
- Assortative mixing coefficient by degree ($A_{r}$): This measure quantifies the tendency observed in networks that nodes with many connections are connected to other nodes with many (or a few) connections [33]. Formally, the coefficient is given by,
  (6) $$A_{r}=\frac{\sum_{ij}A_{ij}\left(k_{i}-\mu \right)\left(k_{j}-\mu \right)}{\sum_{ij}A_{ij}{\left(k_{i}-\mu \right)}^{2}},$$
  where $\mu =\frac{\sum_{ij}A_{ij}k_{i}}{\sum_{ij}A_{ij}}$. For perfectly assortative networks, the coefficient reaches a maximum value of 1, whereas a minimum value of -1 is observed for perfectly dissortative ones.

## 3. Results

We constructed recurrence networks from texts described in https://figshare.com/articles/Recurrence_networks_in_natural_languages/7885376 [34]. The corpus is comprised of 85 books from 17 different languages, corresponding to 4 linguistic families (Germanic, Romance, Slavic, and Uralic). In order toTo validate our method for relatively short sequences, in our calculations we restrict ourselves to segments with 15,000 symbols and repeat the calculations for 5 segments of this length. In our case we have kept the punctuation marks and the space mark as symbols.

**Figure 1:**
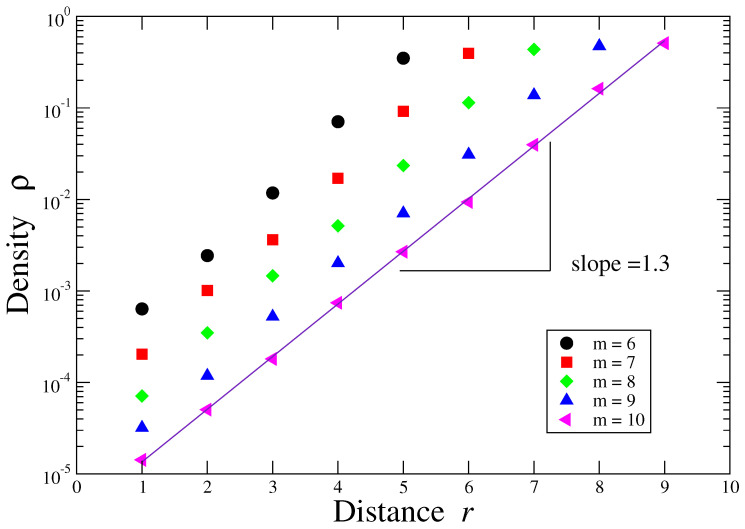
Log-linear plot of density ρ vs. the distance *r* for several values of the pattern length *m*. Here we show the cases m=6,7,8,9,10 and *r* runs from 1 to $r_{max}$, where $r_{max}=m-1$. The fit corresponds to the case m=10, which yields to d≈1.3.

**Figure 2:**
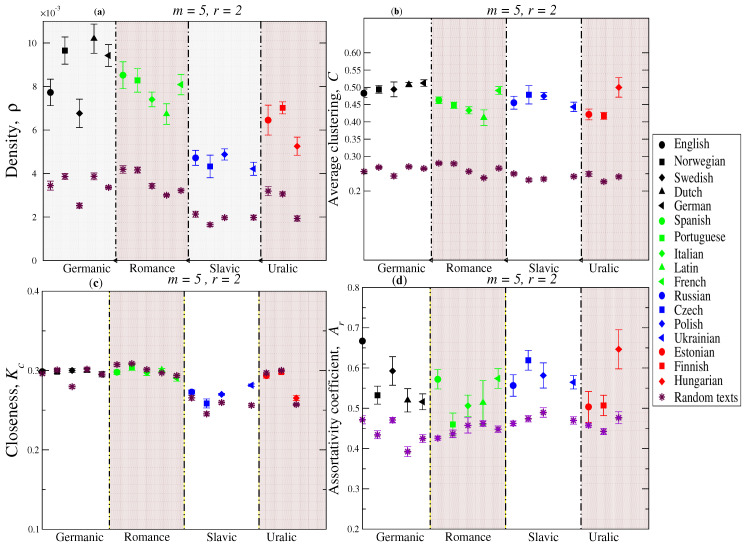
Representative metrics of recurrence-pattern networks for different languages. (**a**) Density for languages grouped by linguistic families. (**b**) Average clustering coefficient C. (**c**) Closeness centrality. (**d**) Assortativity coefficient. Vertical bars indicate the standard deviation of the data.

Prior to the presentation of network metrics results, we explored the behavior of the recurrence-network connectivity in terms of the tolerance parameter *r* for different values of the pattern length *m*. The recurrence rate [35] is a useful measure to quantify the connectivities and it is related to the correlation sum defined in the context of correlation dimension [25] and to the density in the context of networks. We notice that given the length of the alphabet *L* in one natural language, the number of manners that *r*-number of discrepancies that may occur is given by $r_{0}=L^{r}$, where $r_{0}$ represents the total number of permutations with repetitions. Figure 1 shows the behavior between the density ρ and *r* in a log-linear plane for a text in the English language. A linear behavior is observed, where the slope is given by the exponent value d=1.3±0.02 for m=10. Next, we calculated several network metrics for the texts from different languages. As we stated above, in our calculations we considered 5 segments with N= 15,000 elements and we set m=5, which is a value that roughly corresponds to the mean word length in several languages [26,36,37,38]. The threshold error value is set to r=2. The results for the density ρ are presented in Figure 2a, where languages were grouped according to the linguistic family to which they belong. We observe that the Germanic family exhibits high values of density, followed by the Romance family, whereas the Slavic and the Uralic ones are represented by lower values. Here, a high value of density indicates that the number of recurrences is higher than recurrences in other families. For each language, we also generate a surrogate text sequence by shuffling the characters of the original text and show its corresponding value of the density. These results clearly indicate that the Germanic family is the one that is more separated from their corresponding random cases, while both the Slavic and the Uralic are located close to the random configuration.

The results of local structure represented by the clustering coefficient are depicted in Figure 2b. In this case, all the languages exhibit a similar intermediate average value for this measure, suggesting that this value of local structure is likely “universal” across different languages, i.e., the probability that neighbors of a pattern are also neighbors with each other is somehow intermediate. We noticed that for shuffled texts, the values of the average clustering coefficient are smaller (around 0.2) than the values of the original ones. The identification of this intermediate value of the clustering may help to understand the balanced local and global structures.

Moreover, the average closeness values are quite similar across different linguistic families, except data from the Slavic family and Hungarian, which display values below 0.27, indicating that their average farness (inverse distance) between nodes are smaller than values from the other languages, i.e., large distances from a node (pattern) to all other nodes are more likely to observe in recurrence networks from Slavic and Hungarian texts (see Figure 2c). Unlike the clear difference observed between the two previous network metrics and their corresponding shuffled version, the average closeness values obtained from randomized data mostly overlap with original calculations of closeness, confirming that values of shortest paths between nodes almost do not change after randomizing texts. This result points out that both original and random recurrence-pattern networks share one of the recognized small-world properties [39], i.e., while distance between nodes tends to be small in both networks, the clustering (local structure) is higher for original texts (see panels (b) and (c) in Figure 2).

The mixing pattern by degree represents an alternative to capture the tendency of correlations in terms of connectivities. For instance, assortative mixing indicates that there are positive correlations between node’s degree, i.e., nodes with many connections tend to connect to other nodes that also have many connections, while negative correlations (dissortative mixing) indicate that nodes with many connections tend to connect to other nodes that have few few connections. If no correlation by degree is identified, it is said that there is no mixing pattern. The results of mixing pattern are shown in Figure 2d. We found an assortative mixing pattern for all the languages under study with values between 0.5 to 0.7, which are relatively higher than typical values reported for other systems [33,40].

In the same direction, we also evaluated the behavior of the average nearest- neighbor connectivity as a function of the degree.

**Figure 3:**
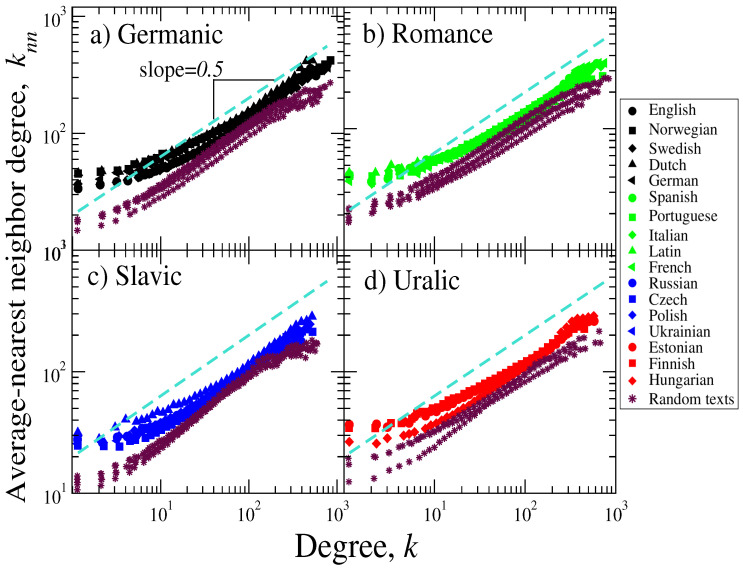
Mean nearest-neighbor connectivity as a function of the degree for (**a**) Germanic, (**b**) Romance, (**c**) Slavic, and (**d**) Uralic linguistic families. For each language, we also show the values of $k_{nn}$ corresponding to shuffled texts. A scaling behavior is observed for all cases of the form $k_{nn}∼k^{\delta }$. We estimate the scaling exponent for degree values 10<k<500, yielding the average values $\bar{\delta }\approx 0.49$ and $\bar{\delta_{r}}\approx 0.47$ for the original and random data, respectively. As a guide for the eye, the dashed line corresponds to the slope = 0.5.

Figure 3 shows the results of the behavior of $k_{nn}$ vs. *k* for original and randomized texts. We observe a scaling behavior for all languages of the form $k_{nn}∼k^{\delta }$, with δ≈0.49±0.03 for original texts, while for random ones, the exponent is δ≈0.47±0.02. It is worth to It is worth remarking that for low low-degree values, the values of $k_{nn}$ from original and random texts are markedly different from each other.

**Figure 4:**
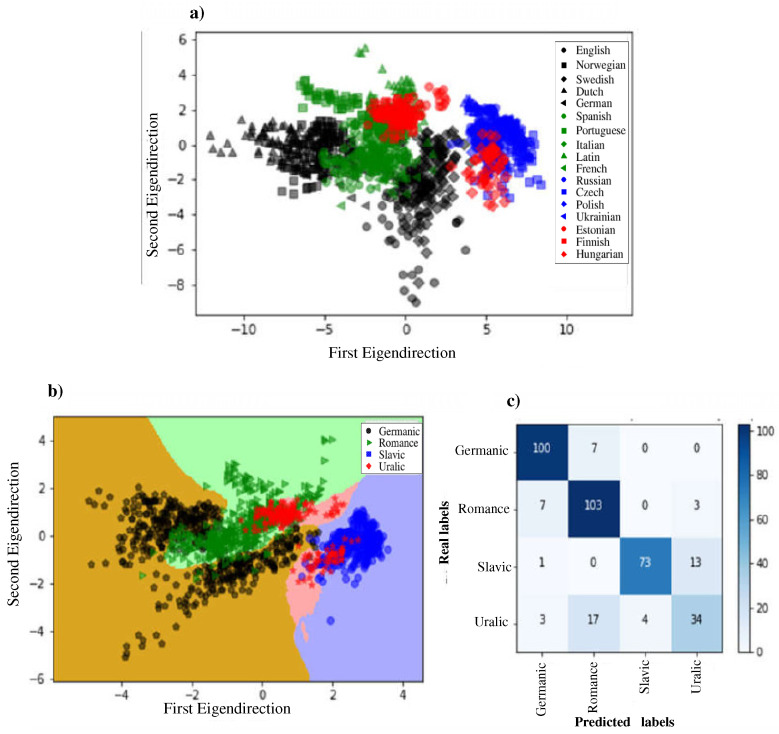
Results of classification analysis applied to European languages. (**a**) Results of the linear discriminant method. Here we show the projection of density values from pattern lengths m=5,6,7. For each *m*-value and for each language, we considered ten segments with length $10^{4}$ to obtain ten ρ values. Next, languages were labeled in classes according to the linguistic family to which they belong (Romance, Germanic, Slavic, Uralic). (**b**) Results of the application of the *k*-nearest- neighbor classification method to data in panel a) but assigning the same label to languages of the same family. We used k=20 neighbors in the classifier. We observe that the families are segregated, except in the case of the Uralic family, which led to two disjoint regions. (**c**) Results of the confusion matrix. The system makes a clear distinction between almost all family languages, except the case of Uralic, where we observe a problem distinguishing this family from Slavic and Romance.

Finally, in order toto provide a direct comparison between different languages based on based on network-metric values, we applied the Fisher’s linear discriminant analysis (LDA) [41] to the density values reported in Figure 2. This technique is very useful to determine if the density could potentially classify languages into the linguistic families they belong to. For this analysis we considered the average density values for each language. Then, the data were projected down to a two-dimensional scatter plot presented in Figure 4a. We observe a separation between clusters formed by languages that belong to the same linguistic family, except the case of the Uralic and Romance families, which are divided into two clusters each one. For a better evaluation of the separation of the clusters provided by the discriminant analysis, we also applied the *k*-nearest- neighbor classification method using as input the results provided by the LDA method. The results are presented in Figure 4b,c. The performance of the system reports that the classifier correctly guessed 89.4% of the times by using m=5,6,7 as input information.

## 4. Discussion and Conclusions

Our results point out that network metrics applied to recurrence dynamics permit to characterizethe characterization of the natural language at different levels. At the local level, metrics like such as clustering coefficient revealed that the local structure is quite similar across different languages, suggesting a general property of organization in natural languages. At the global level, information likesuch as mixing patterns and closeness also indicate that there some similar features, but other metrics like such as the density assign different values to different linguistic families while languages which belong to the same family exhibit similar values. Notably, the profiles of correlations in connectivities of nearest neighbors appear in a similar way across different linguistic families. The results we report here are in general concordance with previous studies focused on phonological networks [42,43,44], which reported positively correlated behavior for assortative mixing and relatively low value for clustering coefficients. In our case, we observed higher values for both mixing coefficient and clustering coefficients compared to the values reported in phonological networks [13,42]. The small-world structure was also observed in the recurrence-language networks. This result is consistent with previous studies which argue that lexical retrieval processes are more “efficient” in the sense that a rapid and robust search is optimal for some network configurations [42,44].

Although our approach is based on based on repetition of patterns without considering specific elements like such as words, and the languages come from a diverse range of linguistic families, the properties of recurrence networks suggest that local and global structures display similarities and differences between languages, opening the possibility of a quantitative evaluation between them. Furthermore, real texts exhibited important differences in the network structure compared to texts obtained from randomizations, i.e., natural languages have more complex structure compared to shuffled sequences. It is also noticeable that for some network-based metrics like such as density, some differences are clearly identified between linguistic familiesfamilies, but additional analyses are needed in this direction. The application of the linear discriminant analysisLDA together with the classification method to the network-based values, revealed that some of the languages are segregated but others are not distinguishable from each other. In summary, our recurrence-network procedure have has revealed additional organizational properties of language, which confirms that there exist similarities in network properties, and some differences emerge between languages that belong to different linguistic families. We remark that these network analysisthese network analyses have not been previously reported for natural languages. Moreover, our results reinforcesreinforce the idea that many aspects of language can be evaluated from a network perspective. Finally, we point out that additional studies are needed to fully characterize the recurrence-network properties of natural language.

## Figures and Tables

**Table 1:** Recurrence symmetric matrix for the beginning of Hamlet’s famous soliloquy: To-be-or-not-to-be. Here N=18 and we set m=3. The resulting matrix has 16 rows and columns.

r=2	To_	o_b	_be	be_	e_o	_or	or_	r_n	_no	not	ot_	t_t	_to	to_	ob_	_be
To_	**1**	0	0	**1**	0	**1**	**1**	0	0	**1**	**1**	**1**	0	**1**	0	0
o_b	0	**1**	0	0	**1**	0	**1**	**1**	0	0	**1**	**1**	0	0	**1**	0
_be	0	0	**1**	0	0	**1**	0	0	**1**	0	0	0	**1**	0	0	**1**
be_	**1**	0	0	**1**	0	0	**1**	0	0	0	**1**	0	0	**1**	0	0
e_o	0	**1**	0	0	**1**	0	0	**1**	**1**	0	0	**1**	**1**	0	**1**	0
_or	0	0	**1**	0	0	**1**	0	0	**1**	**1**	0	0	**1**	**1**	0	**1**
or	**1**	**1**	0	**1**	0	0	**1**	0	0	0	**1**	0	0	**1**	**1**	0
r_n	0	**1**	0	0	**1**	0	0	**1**	0	0	0	**1**	0	0	**1**	0
_no	0	0	**1**	0	**1**	**1**	0	0	**1**	0	0	0	**1**	0	0	**1**
not	**1**	0	0	0	0	**1**	0	0	0	**1**	0	**1**	0	**1**	0	0
ot_	**1**	**1**	0	**1**	0	0	**1**	0	0	0	**1**	0	**1**	**1**	**1**	0
t_t	**1**	**1**	0	0	**1**	0	0	**1**	0	0	0	**1**	0	**1**	**1**	0
_to	0	0	**1**	0	**1**	**1**	0	0	**1**	0	0	0	**1**	0	0	**1**
to_	**1**	0	0	**1**	0	**1**	**1**	0	0	**1**	**1**	**1**	0	**1**	0	0
o_b	0	**1**	0	0	**1**	0	**1**	**1**	0	0	**1**	**1**	0	0	**1**	0
_be	0	0	**1**	0	0	**1**	0	0	**1**	0	0	0	**1**	0	0	**1**
